# Persistent high HTLV-1 proviral load in a patient with complete remission of Adult T-cell leukemia/lymphoma (ATL)

**DOI:** 10.1186/1742-4690-8-S1-A44

**Published:** 2011-06-06

**Authors:** Andrea Mangano, Patricia Costantini, Natalia Altamirano, Griselda De Stefano, Paula Aulicino, Luisa Sen

**Affiliations:** 1 Laboratorio de Biología Celular y Retrovirus, Hospital de Pediatría “J.P. Garrahan”, Ciudad Autónoma de Buenos Aires, Argentina; 2 División de Infectología, Hospital Angel H. Roffo, Ciudad Autónoma de Buenos Aires, Argentina; 3 División de Hematología, Hospital Angel H. Roffo, Ciudad Autónoma de Buenos Aires, Argentina

## 

ATL is a highly aggresive lymphoproliferative disorder caused by HTLV-1. While HTLV-1 proviral load increase with progression of the disease, changes following therapy in ATL have not been well characterized. We present a case of ATL in complete remission with persistent high HTLV-1 proviral load.

A 49-year-ol man was diagnosed as HTLV-1 carrier after a blood donation in 2004. He was born in Buenos Aires, a non-endemic area of Argentina. Four years later, he was diagnosed with T-cell lymphoma but rapidly blast cells were observed in peripheral blood and it was classified as T-cell leukemia . Chemotherapy was started in November 2008 and reached complete remission after 6 cycles. In May 2009, Interferon and zidovudine was started as maintainance therapy. HTLV-1 proviral load were log10 5.5 copies/10^6^ PBMCs and log10 5.3 copies/10^6^ PBMCs in September 2009 and December 2009, respectively. Due to persistent high proviral load, the therapy was changed to pegylated interferon, tenofovir and lamivudine (3TC). Proviral load levels were also high at 4 and 14 months on treatment (log10 5.45 copies/10^6^ PBMCs and log10 5.35 copies/10^6^ PBMCs, respectively).

With the combination of interferon and reverse transcriptase inhibitors for more than one year, the patient is in complete remission, 27 month after the diagnosis, despite the fact that he never even attained reduction of HTLV-1 proviral load.

